# Clinical effectiveness, safety, and viral mutagenicity of oral favipiravir for COVID-19: results from a community-based, open-label, randomized Phase III trial

**DOI:** 10.1128/aac.00054-25

**Published:** 2025-06-24

**Authors:** Matthew Tate, Christopher J. R. Illingworth, Gordon MacGregor, Laura Cunningham, Laura Divers, Elaine McCartney, Lucy Paterson, Caroline Kelly, Ann Shaw, Jonathan S. Perkins, Vanessa Silva, Poppy Holland, Carol Dalton, Samantha Carmichael, Elizabeth Douglas, Pamela Surtees, Janet T. Scott, Colin Berry, Sreenu Vattipally, Ana Da Silva Filipe, Lily Tong, Rory Gunson, GETAFIX trial co-investigators, Claire Rooney, Iain B. McInnes, Robert Jones, Emma Thomson, Kevin G. Blyth

**Affiliations:** 1 Department of Respiratory Medicine, Queen Elizabeth University Hospital 473300, Glasgow, United Kingdom; 2 School of Cancer Sciences, University of Glasgow 3526, Glasgow, United Kingdom; 3 MRC University of Glasgow Centre for Virus Research, University of Glasgow 3526, Glasgow, United Kingdom; 4 Cancer Research UK-Glasgow Clinical Trials Unit, Glasgow, United Kingdom; 5 Glasgow Clinical Research Facility, Queen Elizabeth University Hospital 473300, Glasgow, United Kingdom; 6 BHF Glasgow Cardiovascular Research Centre, University of Glasgow 3526, Glasgow, United Kingdom; 7 School of Cardiovascular and Metabolic Health, University of Glasgow 3526, Glasgow, United Kingdom; 8 West of Scotland Specialist Virology Centre, Glasgow Royal Infirmary 59736, Glasgow, United Kingdom; 9 School of Infection and Immunity, University of Glasgow 3526, Glasgow, United Kingdom; 10 Cancer Research UK Scotland Institute, Glasgow, United Kingdom; IrsiCaixa Institut de Recerca de la Sida, Barcelona, Spain

**Keywords:** COVID-19, antiviral agents, randomized controlled trial

## Abstract

Early community treatment of severe acute respiratory syndrome coronavirus-2 (SARS-CoV-2) infection may reduce severe coronavirus disease (COVID-19) incidence. We evaluated clinical effectiveness, safety, and SARS-CoV-2 mutagenicity of favipiravir, an oral viral RNA polymerase inhibitor. We performed an open-label, community-based, randomized Phase III trial, recruiting non-hospitalized adults with mild COVID-19 (WHO ordinal severity score [OSS] ≤ 3). Positive cases were invited to web-based self-screening within 24 h using public health data. Exclusion criteria included symptoms for >7 days, pregnancy/breastfeeding, severe renal/liver disease, gout, and licensed antiviral eligibility. Participants were randomized 1:1 to 10 days favipiravir (Day 1: 3,600 mg; days 2-10: 1,600 mg) or no additional treatment. The primary endpoint was worst recorded OSS up to and including Day 15 (intention-to-treat). The target recruitment was 302. Secondary endpoints included adverse event (AE) rate to Day 60, time-to-viral clearance (TTVC), time-to-symptom resolution (TTSR), and SARS-CoV-2 sequencing variant rate (≥5% frequency) at Day 15 (registration ISRCTN: 31062548; EudraCT: 2020-001904-41). A total of 68,788 adults were invited, and 302 (0.4%) were subsequently randomized between December 2020 and July 2022 (favipiravir [*n* = 152]: standard care [*n* = 150]). Mean (SD) age was 47.2 (13.2), and 230/302 (76%) were vaccinated. Severe outcomes were infrequent, with no intensive care unit admissions/deaths. There was no difference in the primary endpoint: odds ratio 1.18 (95% confidence interval [CI] 0.63–2.20), TTSR (HR 1.03 [95% CI 0.81–1.31]), or TTVC (HR 1.13 [95% CI 0.65–1.97]). Favipiravir was well tolerated with few AEs but was associated with increased variant frequency, including C-to-U mutations. Community administration of favipiravir for mild COVID-19 was not associated with clinical benefits or safety concerns but was associated with SARS-CoV-2 mutagenicity.

**CLINICAL TRIALS:**

This study is registered with ISRCTN as 31062548 and with EU-CTR as 2020-001904-41.

## INTRODUCTION

Severe acute respiratory syndrome coronavirus-2 (SARS-CoV-2) infection remains as a significant public health concern (1). Widespread vaccination has reduced the incidence of severe coronavirus disease (COVID-19) (2), but infection continues to cause substantial morbidity, particularly in patients with impaired immunity and in communities with poor vaccine uptake (3). This places a premium on the development of additional strategies to minimize the impact of future infection waves (4). Antiviral agents have the potential to limit transmission, reduce symptoms, and prevent progression to severe disease. Several trials have demonstrated the clinical effectiveness of various antiviral agents for non-severe COVID-19 (5), but mostly recruited unvaccinated patients. Trials in vaccinated cohorts are therefore needed. The delivery of these studies is challenging since the known dynamics of SARS-CoV-2 infection predict that antivirals should be started promptly (6), requiring rapid response and early community engagement. This open-label, randomized Phase III trial reports on the clinical effectiveness, safety, and viral mutagenicity of oral favipiravir in a United Kingdom (UK) population with mild SARS-CoV-2 infection and high vaccine coverage. The trial was originally designed in the pre-vaccination era targeting outpatients and inpatients. However, as new knowledge regarding COVID-19 evolved, the protocol was adapted to target community cases only. The trial also reports on the development of a bespoke community engagement and recruitment program, with general implications for the delivery of future early intervention approaches.

Originally developed for influenza, favipiravir is a pyrazinecarboxamide pro-drug (T705) that is phosphorylated intracellularly to a nucleoside analogue (ribofuranosyltriphosphate [T705RTP]) (7), which selectively inhibits viral RNA-dependent RNA polymerase (RDRP). *In vitro*, favipiravir effectively inhibits SARS-CoV-2 infection in Vero E6 cells. Favipiravir has been evaluated in >40 human trials (33 Phase I, three Phase II, and four Phase III trials), mainly in influenza. In these trials, favipiravir demonstrated a favorable safety profile, with a low frequency of mild-to-moderate adverse events (AEs), including asymptomatic elevations in serum uric acid and diarrhea. At the time of the current study’s design (2020), favipiravir had been tested in several small head-to-head studies, including an open-label trial, in which favipiravir (*n* = 35) was associated with a shortened time-to-viral clearance (TTVC: 11 vs 4 days) and improved chest imaging compared to lopinavir/ritonavir (*n* = 45), with both arms also receiving aerosolized interferon-alpha (8). Chen et al. also reported a higher 7-day clinical recovery rate in 116 favipiravir-treated cases compared to 120 arbidol-treated cases (71.4 vs 55.9%) in an open-label, randomized trial (9).

## MATERIALS AND METHODS

### Study design and funding

We performed a community-based, open-label, randomized Phase III trial, called Glasgow Early Treatment Arm Favipiravir (GETAFIX). The trial adhered to CONSORT guidelines and was funded by the Scottish Government Chief Scientist Office Rapid Research in COVID-19 program (COV/GLA/20/03). Favipiravir was provided free of charge by Fujifilm Toyama Chemical Co., Ltd., which had no input to design, conduct, analysis, or reporting. The trial was coordinated by the CRUK Glasgow Clinical Trials Unit supported by infrastructure funding from CRUK (Ref. 25355).

### Favipiravir dose selection

Previous pharmacokinetic studies demonstrate that plasma concentrations above the half maximal effective concentration (EC_50_) for SARS-CoV-2 are achieved with 1,800 mg BD on Day 1 and 800 mg BD thereafter. Earlier studies in Ebola had demonstrated good tolerability at higher doses (6,000 mg on Day 1, 2,400 mg on days 2–10) (10). COVID-19 trials (8, 11) reported prior to the current trial used lower doses (1,600 mg BD on Day 1; 600 mg BD days 2–14) with only modest clinical effects. The dose selected was, therefore, 1,800 mg BD on Day 1, followed by 800 mg BD on days 2–10. This was concordant with recommendations made by the Japanese Association for Infectious Diseases (12).

### Objectives and endpoints

The objectives and endpoints selected aligned with recommendations made by the World Health Organization (WHO) Working Group on the Clinical Characterization and Management of COVID-19 infection in June 2020 (13). These included a measure of patient progression through the health care system (selected as the primary endpoint) and measures of survival and viral burden (secondary and exploratory endpoints, respectively).

#### Primary objective and endpoint

The primary objective was to determine the clinical effectiveness of favipiravir in patients with mild SARS-CoV-2 infection, as defined by the WHO COVID ordinal severity score (OSS) of 2 (symptomatic, independent) or 3 (symptomatic, assistance needed), see Fig. S1. The primary endpoint was clinical status, as defined by self-reported, worst OSS up to and including Day 15 post-randomization, in the intention-to-treat population.

#### Secondary objectives and endpoints

Secondary objectives were to determine the safety of favipiravir and its effect on the intensive care unit (ICU) admission rate and overall survival (OS). Safety was assessed by the AE rate up to and including Day 60. The ICU admission rate was defined by the proportion of patients in each arm with a worst recorded OSS ≥ 7 (hospitalized, intubation, and mechanical ventilation). OS was defined as death from any cause up to and including Day 60.

#### Exploratory objectives and endpoints

Exploratory endpoints included time-to-symptom resolution (TTSR), time-to-viral clearance (TTVC), and SARS-CoV-2 mutagenicity. TTSR was defined by the time (days) from randomization to reach an OSS of ≤1. TTVC was defined by the time (days) to negative SARS-CoV-2 polymerase chain reaction (PCR) testing. For this analysis, PCR Ct values were used as a proxy for viral load, with adjustment for baseline Ct values. PCR was performed using the aqPath COVID-19 CE-IVD RT-PCR Kit (Thermo Fisher) on the Amplitude platform. SARS-CoV-2 mutagenicity was defined by the numbers of genomic variants observed at frequencies of ≥5% in nasopharyngeal swab samples collected at Day 15 from participants in each arm.

### Sample size calculation and assumptions

The study was powered to detect a clinical improvement corresponding to an odds ratio (OR) of 1.95 in the cumulative odds of the worst value of the WHO OSS assessed up to and including Day 15. This is equivalent to the probability of having a better outcome (lower OSS) if allocated to favipiravir of 66% (50% under null hypothesis). By this method, an OR > 1 would favor favipiravir. Initial estimates of statistical power generated during design (December 2020) were revised on two occasions in light of changing epidemiology, vaccination, and severe disease event rate (see *Protocol Amendments*). A sample size of 302 retained >85% power to detect an improvement of this magnitude at the 5%, two-sided level of statistical significance.

### Trial visits and procedures

#### Eligibility criteria and screening

Non-hospitalized adults (≥16 years) with symptomatic SARS-CoV-2 infection were potentially eligible. Positive tests could include PCR tests administered in community testing hubs and self-administered lateral flow tests registered via the National Health Service (NHS) Test and Protect. Potentially eligible patients were invited to initially self-screen on a website created for the trial within 24 h of test positivity using live data shared by Public Health Scotland (PHS). The trial was also advertised on local radio, print, and social media channels.

Potentially eligible patients subsequently attended an in-person screening visit at the Queen Elizabeth University Hospital, NHS Greater Glasgow and Clyde (NHSGGC). Following informed written consent, screening data and tests were collected, allowing the deployment of the following additional inclusion criteria: (i) WHO OSS of 2 or 3; (ii) symptom onset within ≤7 days; (iii) able to provide informed written consent; and (iv) able to swallow oral medication. The exclusion criteria included (i) pregnancy or breastfeeding (a negative pregnancy test was required in women of childbearing potential, who were also required to commit to effective contraception during the trial); (ii) renal impairment requiring renal replacement therapy; (iii) severe hepatic impairment defined as Child-Pugh Grade > A, AST/ALT > 5× the upper limit of normal (ULN) or AST/ALT > 3× ULN and bilirubin > 2× ULN; (iv) gout or hereditary xanthinuria; (v) known hypersensitivity to favipiravir or its metabolites; (vi) unable to discontinue contraindicated concomitant medications; (vii) eligible for licensed anti-viral therapy, as defined by the UK clinical commissioning guidance; and (viii) unsuitable in the opinion of the principal investigator for any other reason.

#### Randomization and masking

Eligible participants were randomized 1:1 to 10 days of oral favipiravir (Day 1: 3,600 mg; days 2–10: 1,600 mg) or no additional treatment. Randomization was performed using a minimization algorithm stratified by (i) age (16–50, 51–70, >70 years), (ii) obesity (body mass index > 30) or hypertension, (iii) OSS (2 vs 3), (iv) vaccination status (vaccination defined as receipt of ≥1 dose of any licensed SARS-CoV-2 vaccine), and (v) gender. Individuals allocated to favipiravir received the medication by courier, with the first dose to be taken on the day of randomization. The allocated arm was not masked to investigators or participants.

#### Visit schedule and follow-up

Following randomization, follow-ups were conducted on days 8, 15, 29, and 60. At every visit, the maximum OSS in the past 24 h and the period between visits were recorded, in addition to AEs, concomitant medications, hospitalization, and vital status. AEs were classified according to NCI-CTCAE v4.03. Following protocol amendment 6, remote follow-up visits became default for all patients (see “Protocol amendments,” below). To maximize data available for safety analyses (including laboratory AEs) and exploratory endpoints regarding TTVC and mutagenicity, limited in-person visit capacity was prioritized for screening/randomization and day 8 and 15 favipiravir arm follow-ups. Since follow-up blood tests and nasopharyngeal (NP) swabs were only collected at in-person follow-ups, imbalance was expected in these data.

#### Nasopharyngeal swabs

Two NP swab samples were collected at all in-person visits, including screening/randomization and subsequent in-person follow-ups. One swab was sent to routine virology for immediate processing and SARS-CoV-2 PCR testing. Over the course of the trial, the baseline samples sent by this route were increasingly utilized for viral genomic sequencing as this became embedded in routine care. This baseline sequencing was used to determine the SARS-CoV-2 genotype. cDNA was synthesized from extracted RNA using LunaScript RT SuperMix (New England Biolabs, MA, USA). SARS-CoV-2 cDNA was amplified using a tiled amplicon approach with ARTIC Network nCoV-2019 primers [v4.1] (Integrated DNA Technologies, IO, USA) to generate material for whole-genome sequencing. Library preparation was carried out using the Illumina COVIDSeq Test (Illumina, CA, USA), and sequencing was performed using P2 reagents (200 cycles) and loaded onto NextSeq2000. If baseline sequencing was not performed, the genotype was inferred from PCR targets. The second swab was immediately frozen at −80°C for later viral genomic sequencing for SARS-CoV-2 mutagenicity testing. This was performed following the completion of recruitment at the MRC Centre for Virus Research, University of Glasgow.

### Statistical analysis plan

Statistical analyses were pre-specified in an *a priori* statistical analysis plan. Efficacy analyses were conducted in the intention-to-treat (ITT) population (all randomized participants). Safety analyses were conducted in the safety population (all participants randomized to favipiravir who received at least one dose and all controls).

#### Primary endpoint analyses

The primary efficacy analysis based on OSS, up to and including Day 15, used cumulative odds ordinal regression with planned adjustment for minimization factors. In cases with missing follow-up data, the most recent OSS was carried forward. Four sensitivity analyses were performed: (i) excluding cases with no follow-up data beyond Day 1; (ii) excluding all Day 1 OSSs since the unexpectedly low incidence of severe disease made the primary endpoint vulnerable to masking of any treatment effect by inclusion of the Day 1 score; (iii) a modified ITT (mITT) analysis to account for treatment allocation failure; this comprised all favipiravir cases who received at least one dose and all control participants with follow-up data; and (iv) excluding the small number of participants recruited as an inpatient during the early weeks of the trial with OSS 4.

Subgroup analyses of the primary endpoint were pre-specified by ethnicity, vaccination status, presence of specific comorbidities (obesity or hypertension), age (≤50 vs >50), and SARS-CoV-2 variant (Alpha vs Delta vs Omicron). Secondary efficacy analyses regarding OS and ICU admissions were planned using Cox regression models, assuming sufficient numbers.

For analyses where convergence was an issue with adjusted models, unadjusted models would be used for reporting, provided that the conclusions drawn were the same.

##### Secondary safety analysis

For the safety analysis, all AEs were recorded and classified according to the NCI-CTAE v4.03. AEs graded ≥1 by ≥10% of participants and laboratory anomalies graded ≥2 by >10% were planned to be tabulated, excluding ‘unrelated’ events. Differences in AE rates between arms would be compared using Mann-Whitney *U* tests.

##### Exploratory analyses regarding TTSR and TTVC

TTSR and TTVC were compared between arms using Cox regression models (right-censored) and presented in Kaplan-Meier plots. TTSR compared median time to an OSS ≤ 1 between arms. TTVC compared time to negative PCR test; this analysis was, therefore, restricted to cases with ≥1 follow-up PCR test. Sensitivity analyses were performed using the mITT for TTSR (to account for allocation failure) and interval-censoring for TTVC.

### SARS-CoV-2 mutation analyses

The different viral sequencing methods used at randomization and follow-up precluded a reliable comparison between baseline and favipiravir-exposed genomes. Mutational events were, therefore, compared in favipiravir-treated cases versus controls in Day 15 follow-up samples. Mutagenesis was assessed in terms of the number of minor variants observed at frequencies ≥5%, as previously reported (14). Only cases with available Day 15 follow-ups were included. Initial read processing was conducted using TrimGalore (15). Reads were then aligned to the Wuhan-Hu-1 reference sequence (GenBank ID: MN908947.3) using bwa (16) and processed with SAMFIRE (17), re-trimming and filtering nucleotides to a PHRED score ≥ 30 before identifying single-locus polymorphisms. Samples were then filtered, retaining only those with a mean coding read depth ≥ 100×. Variant frequencies were calculated relative to the initial consensus sequence. To control for potentially false calls, variants that were polymorphic in >2 Day 1 samples were removed. Statistical comparisons used unpaired *t*-tests.

### Protocol amendments

The evolving knowledge base regarding COVID-19 and the dynamic nature of the NHS pandemic response mandated several protocol amendments. A complete list is provided in the supplement. The changes most relevant to the interpretation of the trial included a revision of the statistical power estimation on two occasions based on updated disease event rates (V5, 18/01/2021 and V8, 17/12/2021), exclusion of inpatients (OSS 4), addition of community case identification using live PHS data, and telephone follow-up being made the default format given limited research nursing resource (V6 11/06/2021), and addition of eligibility for licensed antivirals as an exclusion criterion (V7 16/12/2021).

## RESULTS

### Identification, recruitment, and randomization

**Fig 1:**
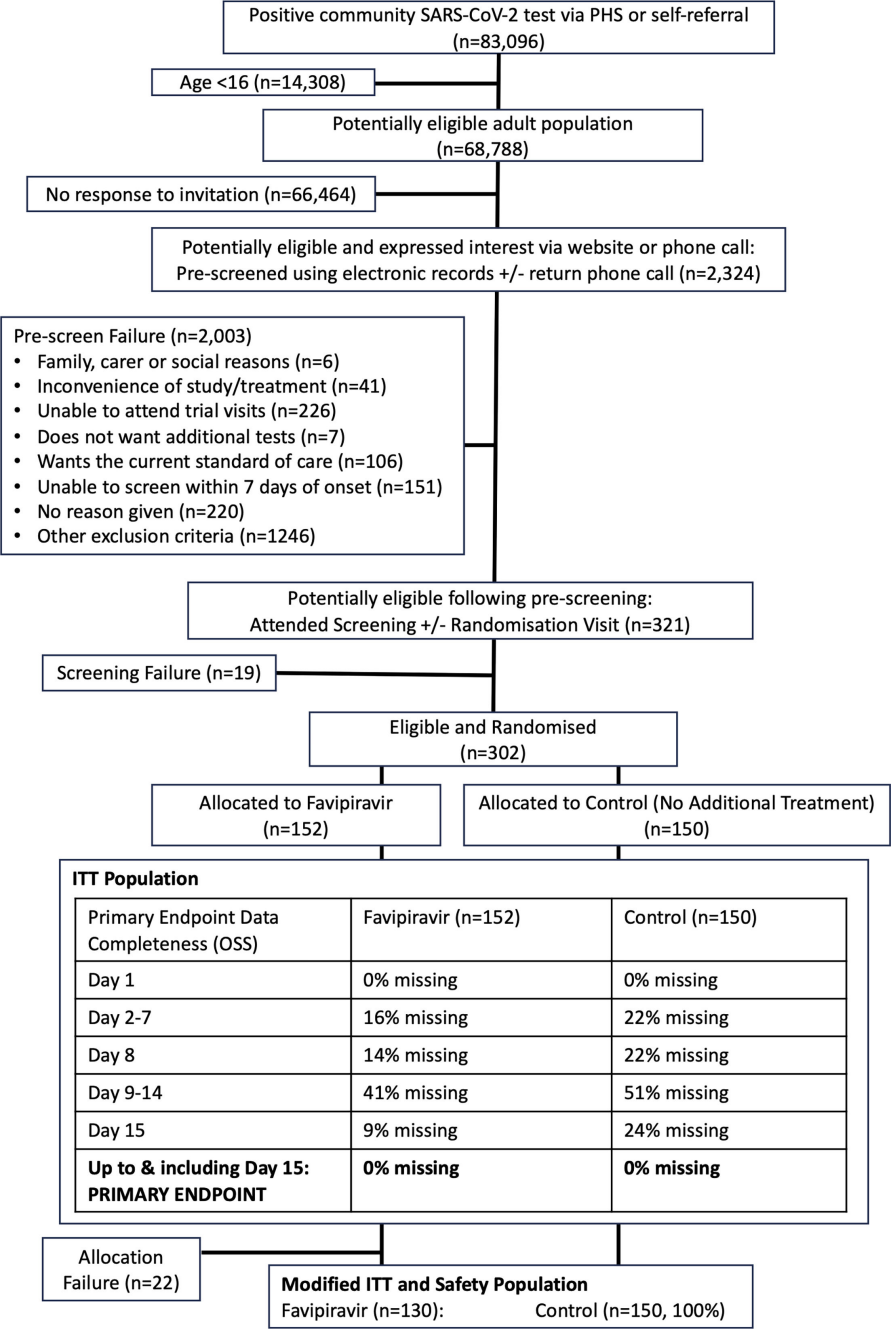
CONSORT diagram summarizing flow through the study from pre-screening to screening, randomization, treatment allocation, and follow-up. The ITT population table details the proportion of cases in each study arm with missing OSS scores at indicated time points.

As summarized in Fig. 1, 83,096 individuals tested positive for SARS-CoV-2 during the recruitment period (December 2020–July 2022). A total of 68,788 (82.7%) were >16 years old and potentially eligible, of whom 2,324 (3.4%) expressed interest via the trial website or phone number. Following pre-screening using electronic health records +/− a follow-up phone call, 321/2,324 (13.8%) patients were invited to a face-to-face screening visit. Following screening, 302 individuals were randomized (favipiravir [*n* = 152]: standard care [*n* = 150]), constituting the ITT population and representing 0.4% of the potentially eligible population (302/68,788). Accordingly, 130/152 (85.5%) participants randomized to favipiravir received ≥1 dose and were included in the safety and mITT populations, alongside all 150 controls. The last follow-up visit for the final participant was completed in September 2022.

### Clinical features at randomization

Baseline clinical features, including age, gender, comorbidities, and vaccination status, were balanced between the arms (see Table 1). The mean age was 47.2 (13.2) years. The most common co-morbidities were asthma (17.2%), hypertension (8.3%), and obesity (5.6%). With this, 64.9% participants reported no significant co-morbidities. The prevalence of smoking was slightly lower in the favipiravir group (11.8% vs 15.3%).

**TABLE 1:** Baseline clinical and COVID-19 illness features^*e*^

Characteristic	Control *N* (%) *n* = 150	Favipiravir *N* (%) *n* = 152	Total *N* (%) *n* = 302
Age group			
16–50	89 (59.3%)	91 (59.9%)	180 (59.6%)
51–70	55 (36.7%)	55 (36.2%)	110 (36.4%)
>70	6 (4.0%)	6 (3.9%)	12 (4.0%)
Gender			
Female	83 (55.3%)	82 (53.9%)	165 (54.6%)
Male	67 (44.7%)	70 (46.1%)	137 (45.4%)
Comorbidities			
Asthma	24 (16.0%)	28 (18.4%)	52 (17.2%)
Other chronic lung diseases	3 (2.0%)	2 (1.3%)	5 (1.7%)
Current smoker	23 (15.3%)	18 (11.8%)	41 (13.6%)
Hypertension	11 (7.3%)	14 (9.2%)	25 (8.3%)
Chronic cardiac disease	1 (0.7%)	5 (3.3%)	6 (2.0%)
Obesity	11 (7.3%)	6 (3.9%)	17 (5.6%)
Depression	5 (3.3%)	8 (5.3%)	13 (4.3%)
Diabetes	5 (3.3%)	4 (2.6%)	9 (3.0%)
Liver disease	3 (2.0%)	5 (3.3%)	8 (2.6%)
Chronic kidney disease	0 (0.0%)	5 (3.3%)	5 (1.7%)
Current malignancy	2 (1.3%)	2 (1.3%)	4 (1.3%)
Other significant comorbidities	9 (6.0%)	4 (2.6%)	13 (4.3%)
No comorbidity	97 (64.7%)	99 (65.1%)	196 (64.9%)
Vaccination status			
Vaccinated	114 (76.0%)	116 (76.3%)	230 (76.2%)
Not vaccinated^*a*^	36 (24.0%)	36 (12.7%)	72 (23.8%)
WHO ordinal severity score (OSS)			
2	129 (86.0%)	136 (89.5%)	265 (87.8%)
3	18 (12.0%)	15 (9.9%)	33 (10.9%)
4	2 (1.3%)	1 (0.7%)	3 (1.0%)
Other^*b*^	1 (0.7%)	0 (0.0%)	1 (0.3%)
COVID-19 illness duration			
Days since first symptom	3.85 (1.16)^*c*^	3.83 (1.09)^*d*^	3.84 (1.12)
Days since last SARS-CoV-2 test	2.99 (0.93)	3.02 (1.03)	3.00 (0.98)
Physiological measurements			
Heart rate (bpm)	80 (12)	79 (12)	80 (12)
Temperature (°C)	36.0 (0.7)	36.0 (0.6)	36.0 (0.7)
Oxygen saturation (%)	98 (2)	98 (2)	98 (2)
SARS-CoV-2 variant			
Alpha	3 (2.0%)	4 (2.6%)	7 (2.3%)
Delta	61 (40.7%)	60 (39.5%)	121 (40.1%)
Omicron	81 (54.0%)	80 (52.6%)	161 (53.3%)
Not categorized	5 (3.3%)	8 (5.3%)	13 (4.3%)

^*a*^ The “Not vaccinated” group includes 70/302 participants (35/150 controls [23.3%], 35/132 [23.0%] favipiravir) recruited prior to licensing of any SARS-CoV-2 vaccine.

^*b*^ In one participant, OSS = 0 was recorded at randomization. This was interpreted as an error since OSS = 0 defines “uninfected; no viral RNA detected,” and the patient had a positive SARS-CoV-2 test.

^*c*^ Not available in 1/150 control cases.

^*d*^ Not available in 1/152 favipiravir cases.

^*e*^ Values are number (%) or mean (SD).

### COVID-19 illness features at randomization

As summarized in Table 1, 298/302 (98.6%) participants were non-hospitalized cases with mild COVID-19, as defined by OSS 2–3. Accordingly, 3/302 (1.0%) were recruited while inpatients (OSS 4) during the initial weeks of the trial. The COVID-19 illness duration was similar between arms, as defined by the symptom duration and the number of days since last test, see Table 1. Baseline viral sequencing was available for 289/302 (95.7%) of participants; PCR targets were used to infer the SARS-CoV-2 genotype in the remainder (18). The distribution of the SARS-CoV-2 genotypes was similar between the groups (see Table 1). The most common genotypes were Omicron BA.2 (29.5%), Delta AY.4 (28.8%), and Omicron BA.1 (16.2%). The prevalence of these variants varied during the trial, consistent with the evolving epidemiology of the UK pandemic.

### Primary objective: clinical status as defined by OSS up to and including Day 15

Based on the ITT population, the median worst OSS up to and including Day 15 was 2 in the treatment arm and 2 in the control arm, see Table 2. The median (range) day on which the worst OSS was recorded was Day 1 (1–15) in the favipiravir arm and Day 1 (1–4) in the control arm. Nineteen of the 302 (6.3%) participants (8/152 [5.3%] favipiravir, 11/150 [7.3%] control) had only Day 1 OSS data recorded (these data were carried forward for primary endpoint analysis, see “Sensitivity analyses,” below [2]).

**TABLE 2:** Worst recorded COVID-19 ordinal severity score (OSS) up to and including Day 15 in favipiravir-treated cases vs controls^*a*^

Worst WHO OSS up to and including Day 15	Control		Favipiravir		Total (all)	
	*N*	%	*N*	%	*N*	%
2	125	83.3	130	85.6	255	84.4
3	23	15.33	20	13.16	43	14.24
4	2	1.33	1	0.66	3	0.99
5	0	0	1	0.66	1	0.33
Total (all)	150	100	152	100	302	100

^*a*^ The median OSS was (2 [IQR = 0]) in both groups, and the odds ratio of a lower OSS in favipiravir-treated cases was not significantly different from 1 (OR 1.18 [95% CI 0.63–2.20]), indicating no effect on clinical status.

In an unadjusted model, the OR of a lower OSS in the favipiravir arm was 1.18 (95% confidence interval [CI] 0.63–2.20), indicating no effect on clinical status. In a model adjusted for minimization factors (age, obesity or hypertension, gender, baseline OSS), the OR remained not significantly different to 1 (1.26 [95% CI 0.53–2.97]).

The treatment effect within each category of each minimization factor was estimated using unadjusted ordinal regression models (Fig. S2).

#### Sensitivity analyses

All sensitivity analyses reported are based on unadjusted models.

##### Exclusion of cases with no OSS beyond Day 1

Nineteen of 302 cases (8/152 [5.3%] favipiravir, 11/150 [7.3%] control) only had Day 1 OSS recorded. When these were excluded, the median (range) day on which the worst OSS was recorded was 1 (1–15) in the favipiravir arm and 1 (1–4) in the control arm, and the OR of a lower OSS in the favipiravir arm was 1.03 (95% CI 0.53–1.99).

##### Exclusion of Day 1 OSS

The low incidence of severe disease meant that in most participants, the highest OSS was recorded on Day 1, with improvement thereafter. This unexpected trajectory made the primary endpoint vulnerable to masking of any treatment effect by inclusion of the Day 1 score. When all Day 1 OSSs were excluded, the OR of a lower OSS in the favipiravir arm remained not significantly different to 1 (0.68 [95% CI 0.42–1.10]).

##### Modified ITT

A total of 130 out of 152 (86%) cases randomized to favipiravir received at least one dose. These cases, plus the 150 controls, constituted the mITT population. The OR of a lower OSS in the favipiravir arm in the mITT population was 1.32 (95% CI 0.68–2.57).

##### Exclusion of cases with OSS 4 at randomization

Three of 302 (1%) were recruited while inpatients and therefore had OSS 4. When these cases were excluded, the OR of a lower OSS in the favipiravir arm was 1.13 (95% CI 0.60–2.14).

### Subgroup analyses

**Fig 2:**
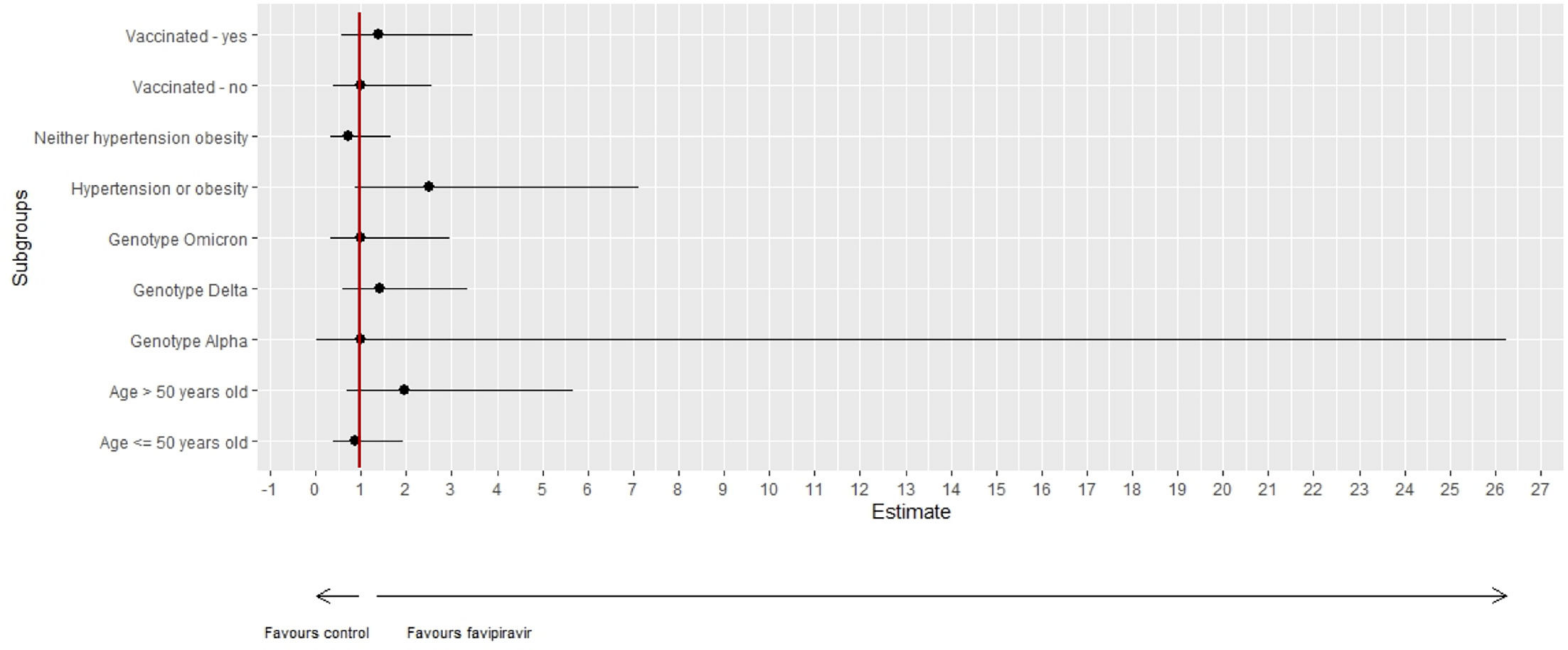
OR in pre-specified sub-group analyses regarding vaccination status, number of comorbidities, age (≤50 vs >50) and SARS-CoV-2 genotype (Alpha vs Delta vs Omicron). The red line refers to an OR = 1.

As summarized in Fig. 2, subgroup analyses for vaccination status, presence of specific comorbidities (obesity or hypertension), age (≤50 vs >50), and SARS-CoV-2 variant (Alpha vs Delta vs Omicron) did not identify any variance in the primary endpoint. The planned ethnicity subgroup analysis was not performed since 298/302 (98.7%) participants were Caucasian.

### Secondary objectives

#### Safety and tolerability

In favipiravir-treated cases, no grade ≥1 AEs or grade ≥2 laboratory events were experienced by >10% of participants. Nevertheless, a complete list of recorded AEs is included in Table S2. No AEs were reported in controls.

The most frequently recorded AEs in favipiravir-treated cases were unspecified ‘general disorders’ (6/130 [4.6%]; all grade 1); dizziness, headache, and eye disorders, all occurring in 2/130 (1.5%). Two of 130 (1.5%) of favipiravir-treated cases discontinued treatment due to side effects both on Day 5: one due to vomiting and abdominal pain and the other due to diarrhea. No other participants reported these AEs (both 1/130, 0.8%). Grade 2 dry eyes, epistaxis, headache, peripheral sensory neuropathy, hyperglycemia, and thrush were recorded in 1/130 favipiravir cases.

#### Serious adverse events

There were eight SAEs recorded (five favipiravir and three control; see Table S3 for details). Of the five SAEs in the favipiravir arm, three were related to favipiravir, and all occurred in a single patient (grade 3 elevated ALT and AST and headache). The other two were grade 3 lung infection and grade 3 sinusitis. All had resolved by Day 60, except for the headache.

#### Laboratory measurements

A complete list of all laboratory AEs is included in Tables S3 and S4. The frequencies of hematological AEs were similar in both arms (Table S4). Biochemical AEs were also similar for most parameters (Table S5). However, grade ≥1 hypercholesterolemia was more common in favipiravir-treated cases than in controls (34.6 vs 19.4%, *P* = 0.003), as was grade ≥1 hypertriglyceridemia (31.5 vs 14.0%, *P* < 0.001) and hyperuricemia (21.5 vs 4.7%, *P* < 0.001).

#### ICU admissions and overall survival

There were no ICU admissions or deaths during the trial precluding this analysis. There were five hospitalizations up to and including Day 60 (three control arm [one COVID-related], two favipiravir arm [two COVID-19-related]). These small numbers preclude any meaningful statistical analysis.

### Exploratory objectives

#### Time-to-symptom resolution (TTSR)

**Fig 3:**
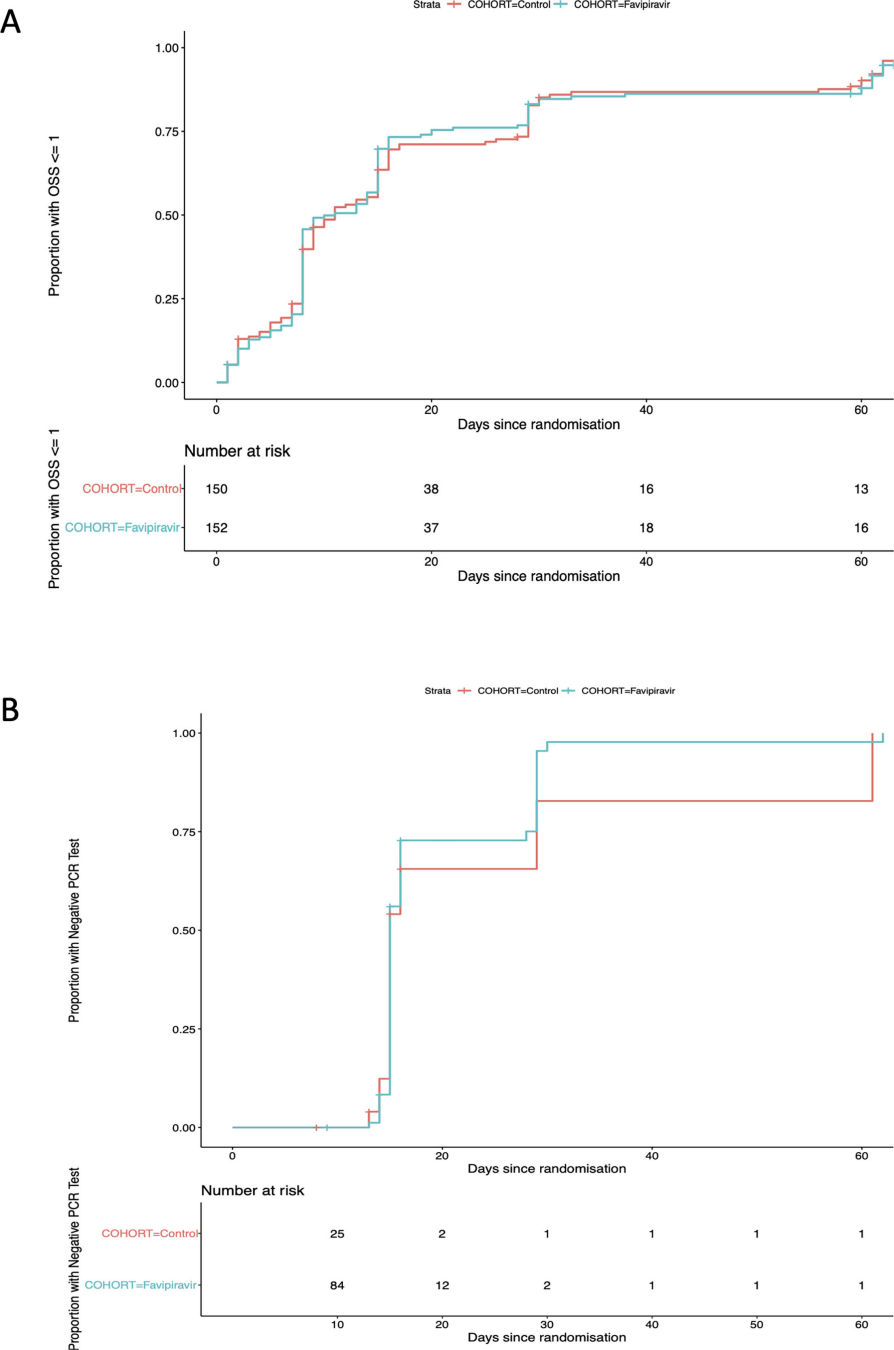
Kaplan-Meier plots summarizing (A) time-to-symptom resolution (TTSR) and (B) time-to-viral clearance (TTVC). A total of 266 out of 302 (88.1%) participants achieved symptom resolution (OSS ≤ 1) by Day 60; there was no difference in TTSR between favipiravir treated cases and controls. A total of 111 out of 302 (36.8%) cases had ≥1 follow-up PCR test and could be included in the TTVC analysis. There was no difference in TTVC between arms.

A total of 266 of 302 (88.1%) participants achieved symptom resolution (OSS ≤ 1) by Day 60. The median (range) TTSR was 11 (1–65) days in the favipiravir arm and 11 (1–68) days in the control arm. As summarized in Fig. 3A, there was no difference in TTSR between arms (HR from unadjusted model 1.03 [95% CI 0.81–1.31]). A sensitivity analysis based on the mITT population to account for allocation failure produced a similar result (HR 1.04 [95% CI 0.81–1.34]).

#### Time-to-viral clearance (TTVC)

A total of 111 out of 302 (36.8%) cases had ≥1 follow-up PCR test and could be included in the TTVC analysis (85 favipiravir, 26 control). The median (range) TTVC was 15 days (13–62) days in the favipiravir arm and 15 (13–61) days in the control arm. There was no difference in TTVC between arms (HR from unadjusted model 1.13 [95% CI 0.65–1.97]), see Fig. 3B. A sensitivity analysis using interval censoring produced a similar result.

#### SARS-CoV-2 mutagenicity

**Fig 4:**
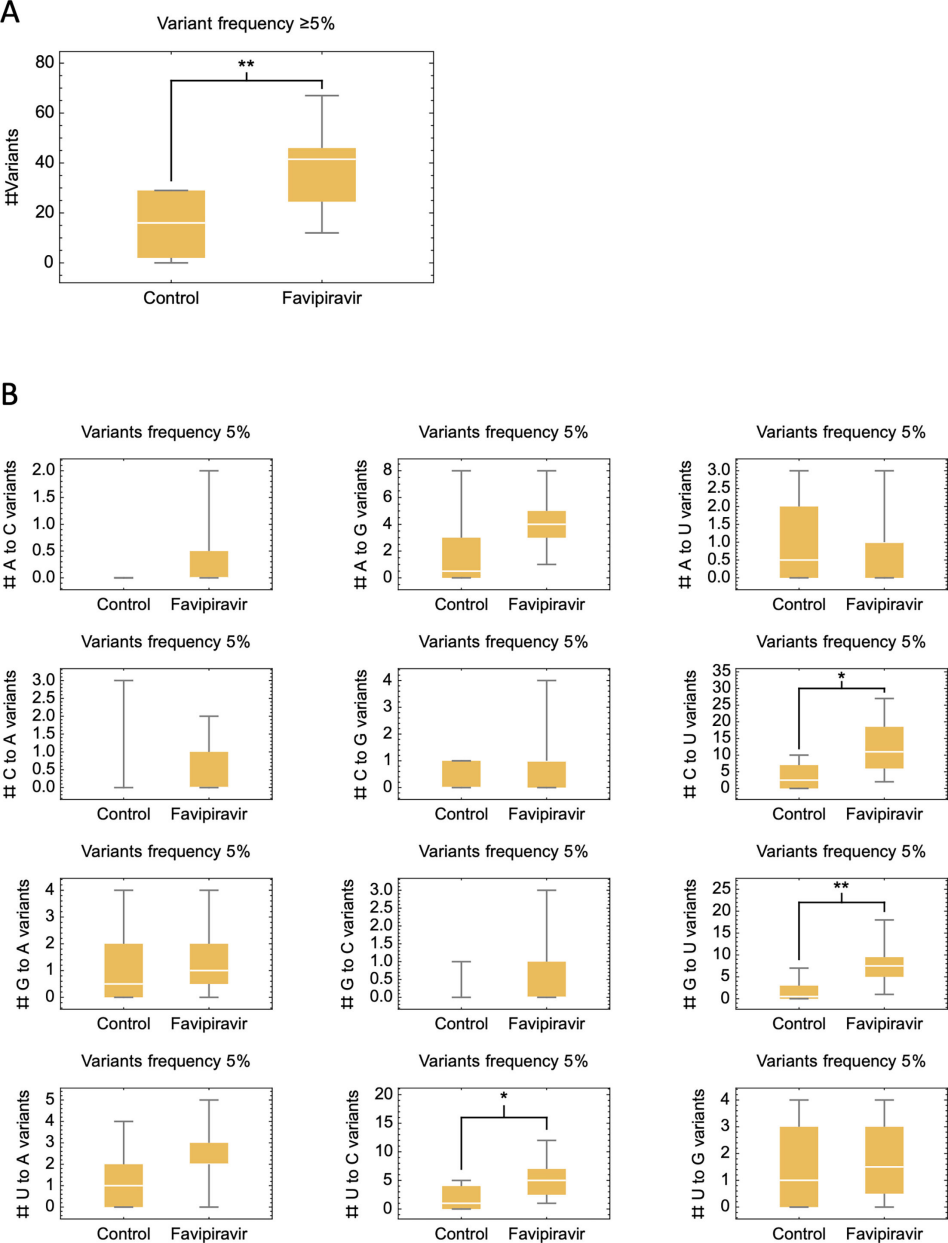
Differences in the SARS-CoV-2 variant frequency (>5%) relative to the initial consensus sequence in Day 15 nasopharyngeal swab samples collected in favipiravir-treated cases versus controls. Significant differences are highlighted by *P < 0.05 and **P < 0.01. (A) Increase in total variant numbers in favipiravir-treated cases. (B) Data for each of the 12 mutational channels (A-to-C, A-to-G, A-to-T, etc.) revealing increases in C-to-U, consistent with the known action of favipiravir, in addition to increases in G-to-U and U-to-C.

Sequencing data were generated from 299 Day 1 samples and 55 Day 15 samples. The baseline samples were used only for data quality assessment, allowing any variants that appeared in >2 samples to be removed as potential false positives. Of the 55 sequenced Day 15 samples, 18 (6.0% of the 302 participants) produced a mean coding read depth ≥ 100× (12 favipiravir, six control) and were, therefore, included in the exploratory analysis of mutational events in favipiravir-treated cases versus controls. As illustrated in Fig. 4A, variant numbers (i.e., the number of positions in the genome with a minor variant at frequency > 5%) were significantly higher in favipiravir-treated cases. Consistent with the known action of favipiravir, a significant increase was also observed in the number of C-to-U variants, see Fig. 4B. No significant difference was observed in the number of G-to-A variants, while significant increases were observed in the numbers of G-to-U and U-to-C variants. Among distinct variants, a single polymorphism in the viral RdRp gene was observed that reached a variant frequency > 50% in one favipiravir-treated case. This is encoded for the non-synonymous variant P809L. Examination of the protein structure demonstrated this variant was not in the active site of the protein (Fig. S3). Additionally, we identified seven variants in the spike protein across both favipiravir and control cases that reached variant frequencies > 50% (Fig. S4). In treated individuals, we identified the mutations P25R, A930D, and G1167C, alongside synonymous mutations at sites 24 and 1116. In controls, we identified synonymous variants at positions 221 and 658. None of the non-synonymous variants found were in the receptor-binding region of the protein. We, thus, did not find clear evidence of gains at consensus level suggesting resistance to favipiravir or novel antigenic function, although the G1167C mutation has been previously noted to occur by convergent evolution in ancestral lineages (19).

## DISCUSSION

In this community-based, open-label Phase III trial, 302 participants with mild COVID-19 were randomized to either 10 days of oral favipiravir or no additional treatment. Despite initiating therapy promptly, we observed no evidence of clinical efficacy based on self-reported worst OSS up to and including Day 15. The incidence of severe disease was considerably lower than expected in both groups, with no ICU admissions, no deaths, and only five hospitalizations. This likely reflected the baseline characteristics of the recruited population, which was relatively young (mean age 47.2 [13.2]) and fit (64.9% reported no comorbidity) and had a high rate of prior vaccination (76.2%). The licensing of other antiviral medications for higher-risk patients during the study period also restricted the eligible population to cases at lower risk of severe disease. Favipiravir was well tolerated with a low rate of treatment discontinuation (1.5%) and a low overall AE rate. We observed no difference in TTSR or TTVC with favipiravir, although our data regarding TTVC are based on limited numbers of repeat samples, particularly in control subjects. SARS-CoV-2 viral genomic sequencing identified evidence of favipiravir-induced mutagenesis, including an increase in C-to-U variants, consistent with the known action of the drug.

Prior to the current trial, several studies reported encouraging data regarding the effect of favipiravir. Chen et al. initially reported accelerated recovery relative to arbidol in patients with moderate disease, although a subsequent report from the same trial showed no effect (11). More recent studies are generally concordant with our findings, including Vaezi et al., who recently reported no difference in the hospitalization rate between favipiravir and placebo in community-treated patients (20). In a recent meta-analysis of nine trials conducted in the outpatient setting, Cheema et al. reported no effect of favipiravir on hospitalization, TTSR, or TTVC. They also found no increase in AE over placebo or no additional therapy (21). Additionally, recent registry outcomes from Japan, where favipiravir is widely available, found no association between the use of the drug and reduced progression to respiratory support (22). Previous influenza (23) and COVID-19 (11, 24) studies have also reported that favipiravir is well tolerated at similar doses, with transient, non-consequential elevations in serum uric acid and liver transaminases being common.

Mutational signatures have recently been associated with exposure to antiviral agents (25), which have the potential to drive the evolution of new SARS-CoV-2 variants. Previous in-vitro studies have demonstrated the mutagenic effect of favipiravir on SARS-CoV-2 (26), although this has not been replicated consistently in vivo (27). In the current trial, viral genomic sequencing from treated cases showed evidence of favipiravir-induced mutagenesis, with increased total variant frequency, including C-to-U variants. However, this result was not unambiguous, with significant increases in other variant types also identified in treated cases. The evolutionary dynamics of within-host evolution are complex, with factors, such as linkage disequilibrium between variants, likely to influence the emergence of higher-frequency nucleotides (28). This analysis is vulnerable to survivorship bias since follow-up samples were collected between days 12 and 15.

The current trial was successful in reaching and initiating therapy quickly in community cases. This required the development of novel methods for raising trial awareness, case identification, and screening. Live public health data were used to maximize study reach, comprising 83,096 positive tests. However, most potentially eligible adults (66,464/68,788, 96.6%) did not respond to email invitations or other means of study advertising, including print, radio, and social media. Of those who did respond, 302/2,324 (12.9%) were subsequently randomized following pre-screening +/− formal screening. This attrition reflects the difficulties involved in delivering trials of this nature and suggests more efficient methods are needed for future trials of early intervention for SARS-CoV-2 infection.

### Study limitations

The incidence of severe COVID-19 disease was lower than expected at initial design, reflecting high adoption of vaccination in the UK. Our findings are not directly applicable to patients at high risk of severe disease, including the elderly, immunosuppressed, and those with multiple comorbidities. Although the primary endpoint was, in theory, robust to changes to the absolute event rate, being based on the overall distribution of worst OSS, the observed distribution implies that a type II statistical error cannot be excluded. Nevertheless, our findings regarding efficacy are concordant with multiple other studies and support the safety and tolerability of favipiravir. Conclusions drawn from viral genome sequence data are limited by the low availability of Day 15 samples. As specified in the Methods section, efforts were made to minimize errors in sequencing analysis, but a larger study with more data points is required to confirm the findings reported here.

### Conclusions

We observed no clinical benefits associated with favipiravir administration in mild COVID-19 in a well-vaccinated UK population with limited comorbidity. Favipiravir was well tolerated but associated with SARS-CoV-2 mutagenesis.

## SUPPLEMENTAL MATERIAL

Supplementary material aac.00054-25-s0001.docx Fig. S1 to S4; Tables S1 to S5.

ASM does not own the copyrights to Supplemental Material that may be linked to, or accessed through, an article. The authors have granted ASM a non-exclusive, world-wide license to publish the Supplemental Material files. Please contact the corresponding author directly for reuse.
