# Supplementary material for: Clinical effectiveness, safety, and viral mutagenicity of oral favipiravir for COVID-19: results from a community-based, open-label, randomized Phase III trial
Source: Antimicrob Agents Chemother. 2025 Jun 24;69(8):e00054-25. doi: 10.1128/aac.00054-25 (PMC12327002; doi:10.1128/aac.00054-25)
Supplement: Supplementary material — Fig. S1 to S4; Tables S1 to S5. [file aac.00054-25-s0001.docx]

**CLINICAL EFFECTIVENESS, SAFETY AND VIRAL MUTAGENICITY OF ORAL FAVIPIRAVIR FOR COVID19: RESULTS FROM A COMMUNITY-BASED, OPEN-LABEL, RANDOMISED, PHASE II/III TRIAL**

**ONLINE ONLY SUPPLEMENT**

**Contents**

Section 1 Figure S1: WHO COVID19 Ordinal Severity Scale (OSS) Page 1

Section 2 Protocol Amendments Page 2

Section 3 Number of OSS scores recorded up to and including day 15 Page 3

Section 4 Figure S2: Treatment Effect within each category of each Page 3

Minimisation Factor via Unadjusted Ordinal Regression

Modelling on OSS up to and Including Day 15

Section 5 Table S1. Complete List of Adverse Events Page 4

Section 6 Table S2. Complete List of Serious Adverse Events Page 5

Section 7 Table S3. Complete List of Haematology Laboratory Adverse Page 6

Events

Section 8 Table S4. Complete List of Biochemistory Laboratory Adverse Page 7-8

Events

Section 9 Fgure S3: Protein Structure of SARS-CoV-2 RdRp in Favipiravir Page 9

treated cases and Controls

Section 10 Figure S4: Protein Structure Of SARS-COV-2 Spike In Favipiravir Page 10

Treated Cases And Controls

**SECTION 1**

**FIGURE S1: WHO COVID19 ORDINAL SEVERITY SCALE**

**SECTION 2**

**PROTOCOL AMENDMENTS**

The rapidly evolving knowledge base regarding COVID19, and the dynamic nature of the NHS pandemic response mandated several amendments to the trial protocol. The following list summarises all amendments made to the original trial protocol (v1, 29/04/2020), including those made after the trial opened in December 2020 using protocol v4, 15/09/2020.

- V2 07/05/2020: adjustment to follow up arrangements after discharge.
- V3 21/07/2020: favipiravir course shortened to 10 days; eligibility extended to any positive COVID-19 test and community cases; risk stratified eligibility added for inpatients; removal of several exclusion criteria
- V4 15/09/2020: Change of Chief Investigator (CI)
- V5 18/01/2021: removal of risk-stratified eligibility for inpatients; simplified outpatient follow-up; SARS-CoV-2 vaccine added to permitted concomitant medication; revised statistical power estimates included based on updated disease event rates
- V6 11/06/2021: Change of CI; Inpatients (OSS 4) removed from eligibility; alignment of inpatient and outpatient schedules; addition of community case identification via PHS data; vaccination added as minimisation factor; telephone follow up as default; revision of baseline blood tests and follow up PCR sampling; further detail regarding concomitant medications.
- V7 16/12/2021: eligibility for licensed antivirals added to exclusion criteria
- V8 17/12/2021: primary outcome timepoint clarified (‘up to and including day 15’ rather than ‘day 15’) ; addition of gout as exclusion criterion; advertising of trial visibility to high-risk groups; provision of envelopes for return of questionnaires; revised concomitant medication detail; revised statistical power estimates included based on updated disease event rates; removal of interim analysis plan.
- V9 18/07/2022: clarification of follow up windows.

| **No. OSS scores recorded up to and including day 15** | **Control** | | **Favipiravir** | | **Total** | |
| --- | --- | --- | --- | --- | --- | --- |
|  | **N** | **%** | **N** | **%** | **N** | **%** |
| **1** | 11 | 7.4 | 8 | 5.3 | 19 | 6.3 |
| **2** | 3 | 2.0 | 2 | 1.3 | 5 | 1.7 |
| **3** | 46 | 30.7 | 19 | 12.5 | 65 | 21.5 |
| **4** | 34 | 22.7 | 46 | 30.3 | 80 | 26.5 |
| **5** | 56 | 37.3 | 77 | 50.7 | 133 | 44.0 |
| **Total (ALL)** | 150 | 100 | 152 | 100 | 302 | 100 |

**SECTION 3**

**TABLE S1: NUMBER OF OSS SCORES RECORDED UP TO AND INCLUDING DAY 15 PER PATIENT, BY STUDY TREATMENT ARM**

**SECTION 4**

**FIGURE S2: TREATMENT EFFECT WITHIN EACH CATEGORY OF EACH MINIMISATION FACTOR VIA UNADJUSTED ORDINAL REGRESSION MODELLING ON OSS UP TO AND INCLUDING DAY 15**

**SECTION 5**

**TABLE S2. COMPLETE LIST OF ADVERSE EVENTS**

**Adverse Events During Screening**

There were 6 AEs reported during screening. 5 were grade 1, reported as “definitely” related to study procedures: raised liver function tests and lightheadedness on the control arm and lightheadedness, syncope and erythema on the favipiravir arm. The remaining event was grade 3 hypertension, unrelated to study procedures, on the favipiravir arm

**Adverse Events on Study**

The table below lists all AEs on study based on the safety population. There were no adverse events reported as having any relationship to study procedures on the control arm. The table therefore only includes data from patients on the Favipirivir arm. Grades 2 or higher have been highlighted in yellow. The worst grade experienced by any patient was grade 3.

|  | **Worst Grade Reported** | | | |
| --- | --- | --- | --- | --- |
|  | **0** | **1** | **2** | **3** |
| Abdominal pain | 129 | 1 | 0 | 0 |
| Alanine aminotransferase increased | 129 | 0 | 0 | 1 |
| Arthralgia | 129 | 1 | 0 | 0 |
| Aspartate aminotransferase increased | 129 | 0 | 0 | 1 |
| Blurred vision | 129 | 1 | 0 | 0 |
| Diarrhea | 129 | 1 | 0 | 0 |
| Dizziness | 128 | 2 | 0 | 0 |
| Dry eye | 129 | 0 | 1 | 0 |
| Dyspepsia | 129 | 1 | 0 | 0 |
| Ear and labyrinth disorders (Other) | 129 | 1 | 0 | 0 |
| Epistaxis | 129 | 0 | 1 | 0 |
| Eye disorders (Other) | 128 | 2 | 0 | 0 |
| Gastroesophageal reflux disease | 129 | 1 | 0 | 0 |
| General disorders and administration site conditions (Other) | 124 | 6 | 0 | 0 |
| Headache | 128 | 1 | 1 | 0 |
| Hyperglycemia | 129 | 0 | 1 | 0 |
| Lethargy | 129 | 1 | 0 | 0 |
| Menorrhagia | 129 | 1 | 0 | 0 |
| Nausea | 129 | 1 | 0 | 0 |
| Nervous system disorders - Other | 129 | 0 | 1 | 0 |
| Peripheral sensory neuropathy | 129 | 0 | 1 | 0 |
| Renal and urinary disorders (Other) | 129 | 1 | 0 | 0 |
| Thrush | 129 | 0 | 1 | 0 |

**SECTION 6**

**TABLE S3. COMPLETE LIST OF SERIOUS ADVERSE EVENTS**

The table below includes all SAEs reported post-randomisation regardless of relatedness to study treatment/procedures. Serious adverse reactions have been highlighted in yellow. All the events were reported as grade 3.

|  |  | Treatment Group | | |
| --- | --- | --- | --- | --- |
|  |  | Control | Favipiravir | Total |
|  |  | n | n | n |
| Infections and infestations | Infections and infestations (Other) | 1 | 0 | 1 |
|  | Lung infection | 0 | 1 | 1 |
|  | Sinusitis | 0 | 1 | 1 |
|  | **Total** | **1** | **2** | **3** |
| Investigations | Alanine aminotransferase increased | 0 | 1 | 1 |
|  | Aspartate aminotransferase increased | 0 | 1 | 1 |
|  | **Total** | **0** | **2** | **2** |
| Nervous system disorders | Heahache | 0 | 1 | 1 |
|  | Total | 0 | 1 | 1 |
| Reproductive system and breast disorders | Pelvic pain | 2 | 0 | 2 |
|  | **Total** | **2** | **0** | **2** |

**SECTION 7**

**TABLE S4. COMPLETE LIST OF HAEMATOLOGY LABORATORY AEs**

The data presented is based on the 280 patients in the safety population. Each parameter was graded according to CTCAE v4.03 and the worst grade experienced derived and tabulated below.

Potential differences between the treatment arms were to be assessed by Mann-Whitney U tests for any parameters affecting more than 10% of patients in either arm at grade 1 or more. There were no such parameters.

|  | | Trial Arm | | | |
| --- | --- | --- | --- | --- | --- |
|  |  | Control | | Favipiravir | |
|  |  | N | % | N | % |
| **Haemoglobin** |  | 149 | 99.33 | 124 | 95.38 |
|  | Grade 0 |  |  |  |  |
|  | Grade 1 | 1 | 0.67 | 6 | 4.62 |
|  | Total (ALL) | 150 | 100.00 | 130 | 100.00 |
| **White Blood Count** |  | 146 | 97.33 | 121 | 93.08 |
|  | Grade 0 |  |  |  |  |
|  | Grade 1 | 4 | 2.67 | 9 | 6.92 |
|  | Total (ALL) | 150 | 100.00 | 130 | 100.00 |
| **Platelets** | Grade 0 | 150 | 100.00 | 128 | 98.46 |
|  | Grade 1 | 0 | 0 | 2 | 1.54 |
|  | Total (ALL) | 150 | 100.00 | 130 | 100.00 |
| **Neutrophils** | Grade 0 | 147 | 98.00 | 124 | 95.38 |
|  | Grade 1 | 3 | 2.00 | 6 | 4.62 |
|  | Total (ALL) | 150 | 100.00 | 130 | 100.00 |
| **Lympocytes** | Grade 0 | 146 | 97.33 | 124 | 95.38 |
|  | Grade 1 | 4 | 2.67 | 4 | 3.08 |
|  | Grade 2 | 0 | 0 | 2 | 1.54 |
|  | Total (ALL) | 150 | 100.00 | 130 | 100.00 |

**SECTION 8**

**TABLE S5. COMPLETE LIST OF BIOCHEMISTORY LABORATORY AEs**

The data presented is based on the 280 patients in the safety population. Each parameter was graded according to CTCAE v4.03 and the worst grade experienced derived and tabulated below. The table below includes all parameters with one or more event at grade 1 or higher.

Potential differences between the treatment arms were to be assessed by Mann-Whitney U tests for any parameters affecting more than 10% of patients in either arm at grade 1 or more. The parameters reaching this threshold are highlighted in yellow and are accompanied by the associated p-value. The distribution of hypercholesteroleamia, hypertriglyceridaemia and hyperuricaemia was significantly different at the 5% significance level, with higher frequencies observed in the favipiravir arm.

|  | | COHORT | | | |
| --- | --- | --- | --- | --- | --- |
|  |  | Control | | Favipiravir | |
|  |  | N | % | N | % |
| Albumin | Grade 0 | 147 | 98.0 | 126 | 96.9 |
|  | Grade 1 | 1 | 0.7 | 4 | 3.1 |
|  | Grade 2 | 2 | 1.3 | 0 | 0.0 |
|  | Total | 150 | 100.0 | 130 | 100.0 |
| ALP | Grade 0 | 149 | 99.3 | 128 | 98.5 |
|  | Grade 1 | 1 | 0.7 | 2 | 1.5 |
|  | Total | 150 | 100.0 | 130 | 100.0 |
| ALT | Grade 0 | 141 | 94.0 | 121 | 93.1 |
|  | Grade 1 | 9 | 6.0 | 9 | 6.9 |
|  | Total | 150 | 100.0 | 130 | 100.0 |
| AST | Grade 0 | 145 | 96.7 | 124 | 95.4 |
|  | Grade 1 | 5 | 3.3 | 6 | 4.6 |
|  | Total | 150 | 100.0 | 130 | 100.0 |
| Bilirubin | Grade 0 | 146 | 97.3 | 123 | 94.6 |
|  | Grade 1 | 2 | 1.3 | 7 | 5.4 |
|  | Grade 2 | 2 | 1.3 | 0 | 0.0 |
|  | Total | 150 | 100.0 | 130 | 100.0 |
| Creatinine | Grade 0 | 150 | 100.0 | 129 | 99.2 |
|  | Grade 1 | 0 | 0.0 | 1 | 0.8 |
|  | Total | 150 | 100.0 | 130 | 100.0 |
| Potassium (high) | Grade 0 | 149 | 99.3 | 129 | 99.2 |
|  | Grade 1 | 1 | 0.7 | 0 | 0.0 |
|  | Cannot be graded | 0 | 0.0 | 1 | 0.8 |
|  | Total | 150 | 100.0 | 130 | 100.0 |
| Potassium (low) | Grade 0 | 150 | 100.0 | 127 | 97.7 |
|  | Grade 1 | 0 | 0.0 | 2 | 1.5 |
|  | Cannot be graded | 0 | 0.0 | 1 | 0.8 |
|  | Total | 150 | 100.0 | 130 | 100.0 |
| Sodium (low) | Grade 0 | 149 | 99.3 | 130 | 100.0 |
|  | Grade 1 | 1 | 0.7 | 0 | 0.0 |
|  | Total | 150 | 100.0 | 130 | 100.0 |
| Cholesterol (High) (p=0.003) | Grade 0 | 119 | 79.3 | 81 | 62.3 |
|  | Grade 1 | 27 | 18.0 | 42 | 32.3 |
|  | Grade 2 | 1 | 0.7 | 3 | 2.3 |
|  | Grade 3 | 0 | 0 | 0 | 0 |
|  | Grade 4 | 1 | 0.7 | 0 | 0 |
|  | Cannot be graded | 2 | 1.3 | 4 | 3.1 |
|  | Total | 150 | 100.0 | 130 | 100.0 |
| Triglycerides (High) (p<0.001) | Grade 0 | 127 | 84.7 | 85 | 65.4 |
|  | Grade 1 | 19 | 12.7 | 34 | 26.2 |
|  | Grade 2 | 1 | 0.7 | 6 | 4.6 |
|  | Grade 3 | 1 | 0.7 | 1 | 0.8 |
|  | Grade 4 | 0 | 0 | 0 | 0 |
|  | Cannot be graded | 2 | 1.3 | 4 | 3.1 |
|  | Total | 150 | 100.0 | 130 | 100.0 |
| Uric Acid (High) (p<0.001) | Grade 0 | 46 | 30.7 | 74 | 56.9 |
|  | Grade 1 | 7 | 4.7 | 28 | 21.5 |
|  | Grade 2 | 0 | 0 | 0 | 0 |
|  | Grade 3 | 0 | 0 | 0 | 0 |
|  | Grade 4 | 0 | 0 | 0 | 0 |
|  | Cannot be graded | 97 | 64.7 | 28 | 21.5 |
|  | Total | 150 | 100.0 | 130 | 100.0 |

**SECTION 9**

**FIGURE S3: PROTEIN STRUCTURE OF SARS-COV-2 RDRP IN FAVIPIRAVIR TREATED CASES AND CONTROLS**

Among distinct variants, we identified a single polymorphism in the viral RNA-dependent RNA polymerase (RdRp) gene that reached a variant frequency >50% in one of the treated individuals. This encoded for the nonsynonymous variant P809L. Examination of the protein structure demonstrated that this variant (in red, below) was not in the active site of the protein. The structure is taken from the Protein Data Bank. The RdRp structure is 6M71 (Ref: Gao, Y. et al. Structure of the RNA-dependent RNA polymerase from COVID-19 virus. Science 368, 779–782 (2020))

**
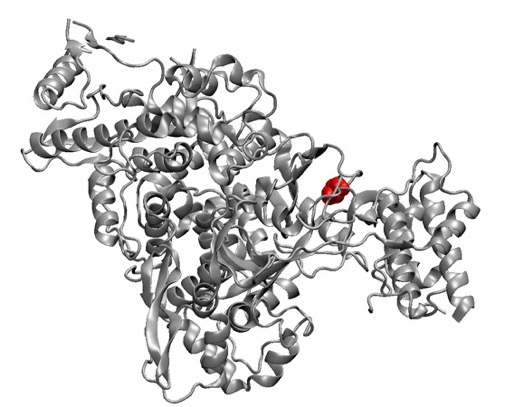
**

**SECTION 10**

**FIGURE S4: PROTEIN STRUCTURE OF SARS-COV-2 SPIKE IN FAVIPIRAVIR TREATED CASES AND CONTROLS**

We identified seven variants in the Spike protein that reached variant frequencies >50%. In treated individuals we identified the mutations P25R, A930D, and G1167C, alongside synonymous mutations at sites 24 and 1116 of the protein sequence. In control individuals we identified synonymous variants at positions 221 and 658 of the protein sequence. Nonsynonymous changes in the proteins are shown in red vdW representation, with synonymous variants in blue. None of the nonsynonymous variants found were in the binding region of the protein. The structure is taken from the Protein Data Bank. The Spike structure is 6VXX (Ref: Walls, A. C. et al. Structure, Function, and Antigenicity of the SARS-CoV-2 Spike Glycoprotein. Cell 181, 281-292.e6 (2020)).

**
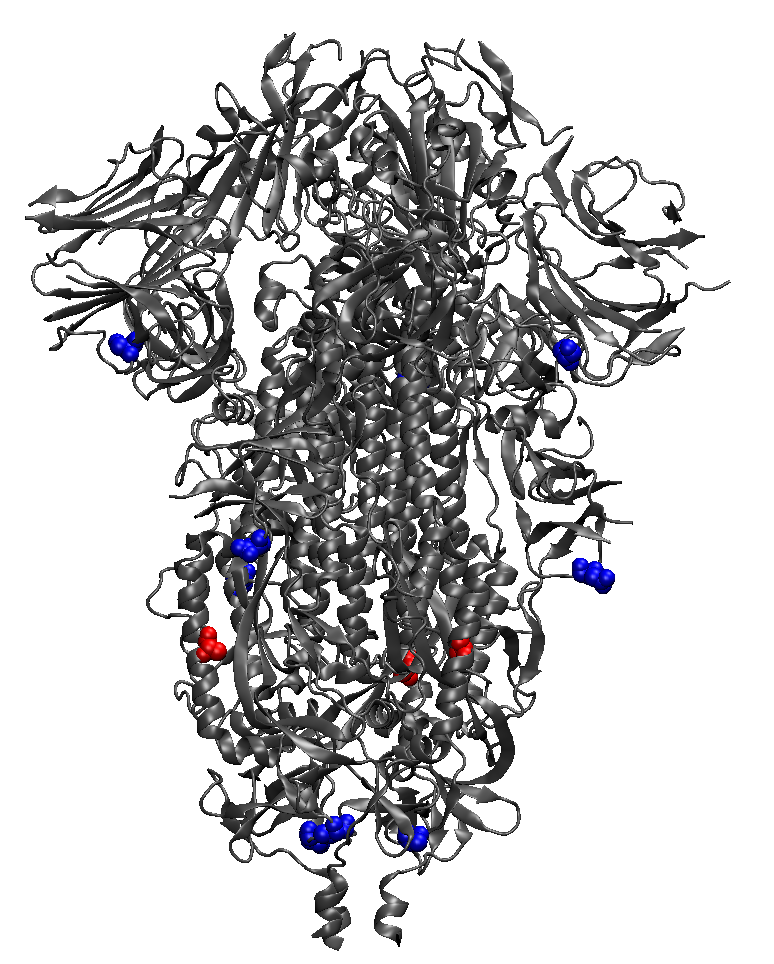
**
